# The ratio of pre-dialysis vancomycin trough serum concentration to minimum inhibitory concentration is associated with treatment outcomes in methicillin-resistant *Staphylococcus aureus* bacteremia

**DOI:** 10.1371/journal.pone.0193585

**Published:** 2018-03-05

**Authors:** Chien-Fang Fu, Jiun-Da Huang, Jann-Tay Wang, Shu-Wen Lin, Chien-Chih Wu

**Affiliations:** 1 School of Pharmacy, College of Medicine, National Taiwan University, Taipei, Taiwan; 2 Department of Internal Medicine, National Taiwan University Hospital, Taipei, Taiwan; 3 Department of Pharmacy, National Taiwan University Hospital, Taipei, Taiwan; 4 Graduate Institute of Clinical Pharmacy, College of Medicine, National Taiwan University, Taipei, Taiwan; Universidade Estadual Paulista Julio de Mesquita Filho, BRAZIL

## Abstract

**Background:**

Vancomycin is a standard treatment for methicillin-resistant *Staphylococcus aureus* (MRSA) bacteremia, and its efficacy is closely linked to the recommended serum trough concentration of 15–20 mg/L. However, it is unknown how the pre-dialysis trough serum concentration (C_pre-HD_) correlates with MRSA eradication in renal failure patients undergoing intermittent hemodialysis (HD).

**Objective:**

To evaluate the relationship between C_pre-HD_ and the treatment outcomes in this population.

**Materials and methods:**

A retrospective study was conducted to enroll renal failure patients undergoing HD who had received vancomycin treatment for MRSA bacteremia during January 2013 to June 2016. Treatment failure was defined as persistent bacteremia after ≥ 7 days of vancomycin therapy or recurrent MRSA infection within 30 days. Patient characteristics, vancomycin dosing regimen, C_pre-HD_, vancomycin minimum inhibitory concentration (MIC), and subsequent culture data were reviewed. The receiver operating characteristic (ROC) curve was used to find the optimal cut-off point of C_pre-HD_.

**Results:**

42 patients were enrolled and 64% had treatment failure. Although there were no significant differences in demographics or C_pre-HD_ between the two groups, C_pre-HD_/MIC was significantly higher in the success group than that in the failure group (22.80±10.90 vs. 14.94±6.11, *p* = 0.019). The area under the ROC curve was 0.74, while the sensitivity, specificity, positive predictive value, and negative predictive value were 67%, 78%, 62.5%, and 81%, respectively, at the optimal C_pre-HD_/MIC of ≧ 18.6.

**Conclusions:**

C_pre-HD_/MIC was associated with vancomycin treatment outcome in MRSA bacteremia, and targeting to achieve a C_pre-HD_/MIC of ≧ 18.6 may improve treatment outcomes in renal failure patients who are on intermittent HD.

## Introduction

Methicillin-resistant *Staphylococcus aureus* (MRSA) is a common pathogen which causes infections, especially among patients who undergo dialysis.[1] Vancomycin is an antibiotic glycopeptide which kills bacteria by inhibiting cell wall synthesis, and it is the first line treatment for MRSA infection if minimum inhibitory concentration (MIC) is lower than 1 mg/L [2]. The clinical efficacy of vancomycin is best predicted by the area under the curve over 24 hours (AUC_24_) to the MIC ratio, and a target AUC_24_/MIC ratio of ≥ 400 is recommended by the Infectious Diseases Society of America (IDSA) Practice Guideline[2–4]. The trough serum concentration (C_trough_) of vancomycin is recommended as a surrogate for the AUC_24_ and should be maintained between 15–20 mg/L to achieve an AUC_24_/MIC ≥ 400 when MIC is ≤ 1 mg/L[2, 5]. However, among previous studies that investigated the association between vancomycin AUC_24_/MIC or C_trough_ and treatment outcome, few examined renal failure patients who received renal replacement therapy such as hemodialysis (HD)[3, 4, 6, 7]. The immune function was changed in renal failure patients, which may affect treatment outcome than that in general population.[8] Therefore, the AUC_24_/MIC or C_trough_ target may be different in this vulnerable population. As a result, current vancomycin dosing regimen for HD patients relies mainly on extrapolation or modeling.[9] Although pre- or post-HD serum concentration monitoring has been adopted as a common clinical practice for years in vancomycin-treated patients with renal failure under HD, the best time of sampling remains controversial[10]. Moreover, previous studies all focused on developing specific dosing protocols in renal failure patients to achieve the target vancomycin serum concentration of 10–20 mg/L[11–15]. Nonetheless, evidence is still lacking to show any correlation between pre-dialysis vancomycin trough serum concentration (C_pre-HD_) and the clinical outcomes in this patient population. Our aim is to investigate the relationship between C_pre-HD_ and treatment outcomes of MRSA bacteremia in patients undergoing HD.

## Materials and methods

This retrospective study was conducted in the National Taiwan University Hospital (NTUH), which is a tertiary medical center with a 2600-bed capacity in northern Taiwan., and patients profiles during January 2013 to June 2016 of the Integrated Medical Database (NTUH-iMD) was used for patient enrollment. This study had been approved by the Research Ethics Committee of NTUH (201602044RINB). Adult patients (≥ 20 yrs) with chronic renal failure undergoing HD three times per week were included if they had documented MRSA bacteremia, and received vancomycin treatment within 72 hours after the blood sample from which the MRSA was first isolated. MRSA bacteremia was defined as at least one set of blood culture that was collected when systemic inflammatory response syndrome was present and yielded MRSA. Patients were excluded for missing C_pre-HD_ measurements during the treatment course, vancomycin use less than a week, absence of following blood cultures collected after vancomycin treatment, ever receiving other antibiotics for MRSA treatment (such as daptomycin, linezolid, teicoplanin and tigecycline) within 7 days before vancomycin treatment, receiving continuous renal replacement therapy (CRRT) during vancomycin treatment or daily urine output over than 400 mL.

The dosing regimen of vancomycin for patients under HD in our institution was 15–20 mg/kg as a loading dose (LD), followed by 7–10 mg/kg after each HD as maintenance doses (MD). The sampling time of C_pre-HD_ was within one hour before the third HD session since giving one LD and two MD. The vancomycin serum concentrations were measured by a chemiluminescent microparticle immunoassay (ARCHITECT i2000SR immunoassay analyzer, Abbott Laboratories, Abbott Park, Illinois, USA). The limit of detection (sensitivity) was 0.24 mg/L, and the coefficients of variation at 7.0, 35.0, and 75.0 mg/L were < 5%. For hemodialysis, the blood and dialysate flow rates were set at 200 and 500 mL/min, respectively. The dialysis lasted 4 hour each time and the dialyzers used were F6HPS, FX60, FX80, or FX100 by nephrologist’s decision (Fresenius Medical Care, Bad Homburg, Germany).

A standardized case report form was used to collected data including demographic characteristics (sex, age, height, and weight), the Charlson Comorbidity Index (CCI) and underlying diseases, sources and sites of infection (based on medical records and culture sampling sites), subsequent blood cultures results, immunosuppressant usage (i.e. prednisolone ≥ 10mg/day or other corticosteroids of equivalent potency, calcineurin inhibitors, mechanistic target of rapamycin (mTOR) inhibitors, azathioprine, and mycophenolate mofetil), catheterization, catheter removal, vancomycin doses and duration of treatment, C_pre-HD_, MIC of vancomycin for MRSA, concurrent antibiotics usage, shock status (use of vasopressors or inotropics), and 30-day mortality. The MIC of vancomycin for the first isolate was determined by the broth microdilution method according to the Guidelines of the Clinical and Laboratory Standards Institute (CLSI) except for two MIC of vancomycin that was determined by E-test according to the manufacturer’s instruction.[16] Because AUC_24_ and C_pre-HD_ are highly correlated in patients receiving HD, we calculate C_pre-HD_/MIC as a surrogate of AUC_24_/MIC to evaluate its effect on treating MRSA bacteremia.[9]

Treatment failure was defined as persistent bacteremia after ≥ 7 days of vancomycin treatment, or recurrent MRSA infection within 30 days after the first negative conversion of blood culture.[2]

Data were described as mean ± standard deviation or number with percentage. The two-tailed independent *t* test was used for continuous data, and either Chi-square or Fisher’s exact test was used for categorical data. A *p*-value of 0.05 or lower was considered statistically significant. The receiver operating characteristic (ROC) curve was used to determine the optimal cut-off point of C_pre-HD_/MIC, which had the maximum Youden index. The statistical analysis was performed by SPSS 18 (SPSS Inc, Chicago, Illinois, USA).

## Results

Sixty-five HD patients who received vancomycin due to MRSA bacteremia were initially enrolled. Twenty-three patients were excluded for lacking following blood culture collected (n = 8), missing pre-dialysis serum level data (n = 3), using other antibiotics within 7 days before vancomycin treatment (n = 7), or receiving CRRT (n = 5) during vancomycin treatment. Forty-two patients were available for the final analysis.

Fifteen patients (36%) were successfully treated, and the other 27 were classified as treatment failure (64%). In the treatment failure group, 25 patients had persistent bacteremia, and 2 had recurrent MRSA infection, which two weeks course of antibiotics was completed before. Although there were no significant differences in baseline demographics, underlying disease, sources of bacteremia, or vancomycin regimens between the success and failure group, more patients received immunosuppressants in treatment failure group (Table 1). Catheters were all removed in both groups after confirming it as infection source. C_pre-HD_ values were not significantly different between the success and failure groups (17.40 ± 4.55 vs. 15.22 ± 4.24 mg/L, *p* = 0.139) (Table 2). However, C_pre-HD_/MIC was significantly higher in the success group compared to that of the failure group (22.80 ± 10.90 vs. 14.94 ± 6.11, *p* = 0.019). There was also no significant difference in 30-day mortalities between the success and failure groups (11% vs 19%, *p* = 0.666).

**Table 1:** Clinical characteristics and vancomycin dose, classified by vancomycin treatment outcomes.

	Total patients (N = 42)	Success group (N = 15)	Failure group (N = 27)	*p* value
Age	69.00 ± 14.81	71.80 ± 13.23	67.44 ± 15.63	0.346
Height (cm) (mean ± standard deviation)	159.57 ± 8.10	160.09 ± 6.83	159.29 ± 8.84	0.747
Weight (kg) (mean ± standard deviation)	56.23 ± 9.597	59.25 ± 9.66	54.55 ± 9.32	0.137
Male sex	20 (48)	5 (33)	15 (56)	0.209
Underlying disease				
Charlson Comorbidity Index (mean ± SD)	4.71 ± 2.04	4.53 ± 1.51	4.81 ± 2.30	0.636
Cardiovascular disease	28 (67)	12 (80)	16 (59)	0.306
Chronic liver disease	4 (10)	2 (13)	2 (7)	0.608
Diabetes mellitus	21 (50)	8 (53)	13 (48)	1.000
Malignancy	5 (12)	0 (0)	5 (19)	0.142
Neurological disease	9 (21)	2 (13)	7 (26)	0.451
Peptic ulcer disease	14 (33)	5 (33)	9 (33)	1.000
Connective tissue disease	1 (2)	0 (0)	1 (4)	1.000
Neutropenia	0 (0)	0 (0)	0 (0)	
Septic shock	14 (33)	5 (33)	9 (33)	1.000
Polymicrobial bacteremia	11 (26)	3 (20)	8 (30)	0.717
*Acinetobacter baumannii*	2 (5)	1 (7)	1 (4)	
*Burkholderia cepacia* complex	1 (2)	1 (7)	0 (0)	
*Enterococcus faecalis*	2 (5)	1 (7)	1 (4)	
*Enterococcus faecium*	1 (2)	0 (0)	1 (4)	
*Escherichia coli*	3 (7)	1 (7)	2 (7)	
*Klebsiella pneumoniae*	3 (7)	0 (0)	3 (11)	
*Pseudomonas aeruginosa*	1 (2)	0 (0)	1 (4)	
*Serratia marcescens*	1 (2)	1 (7)	0 (0)	
*Stenotrophomonas maltophilia*	1 (2)	0 (0)	1 (4)	
Immunosuppressants use	6 (14)	0 (0)	6 (22)	0.056
MRSA bacteremia source				0.981
Bone and joint	0 (0)	0 (0)	0 (0)	
Catheter-related	11 (26)	4 (27)	7 (26)	
Endocarditis	2 (5)	0 (0)	2 (7)	
Pneumonia	6 (14)	2 (13)	4 (15)	
Surgical wound or skin and soft tissue	6 (14)	2 (13)	4 (15)	
Unknown	17 (41)	7 (47)	10 (37)	
Vancomycin MIC (mg/L) (mean ± SD)				0.075
< 0.5	7 (16)	5 (33)	2 (7)	
1	29 (69)	9 (60)	20 (74)	
1.5	2 (5)	1 (7)	1 (4)	
2	4 (10)	0 (0)	4 (15)	
Concurrent antibiotics	31 (74)	10 (67)	21 (78)	0.481
Penicillin group	6 (14)	2 (13)	4 (15)	
Cephalosporin group	24 (57)	6 (40)	18 (67)	
Carbapenem group	8 (19)	1 (7)	7 (26)	
Fluoroquinolone	1 (2)	0 (0)	1 (4)	
Trimethoprim/sulfamethoxazole	3 (7)	2 (13)	1 (4)	
Aminoglycoside	1 (2)	0 (0)	1 (4)	
Colistin	1 (2)	0 (0)	1 (4)	
Antifungal agents	1 (2)	0 (0)	1 (4)	
Antiviral agents	2 (5)	0 (0)	2 (7)	

SD: standard deviation. MRSA: methicillin-resistant *Staphylococcus aureus*.

**Table 2:** Vancomycin dosing profile, serum concentrations, and 30-day mortalities in total, success, and failure groups.

	Total patients (N = 42)	Success group (N = 15)	Failure group (N = 27)	*p* value
Vancomycin dosage				
Loading dose (mg/kg) (mean ± SD)	17.95±5.97	17.29±4.02	18.32±6.86	0.546
Maintenance dose (mg/kg) (mean ± SD)	8.54±1.71	8.55±2.15	8.54±1.45	0.992
C_pre-HD_ (mean ± SD)	16.00±4.43	17.40±4.55	15.22±4.24	0.139
≥ 15 mg/L	26 (62)	11 (73)	15 (56)	0.330
C_pre-HD_/MIC ratio (mean ± SD)	17.75±8.88	22.80±10.90	14.94±6.11	0.019*
≥ 18.6	16 (38)	10 (67)	6 (22)	0.008*
30-days mortality	7(17)	2(13)	5(19)	1.000

SD: standard deviation. C_pre-HD_: pre-dialysis trough serum concentration. MIC: minimum inhibitory concentration.

* p<0.05

In the treatment failure group, the antibiotic agent was switched from vancomycin to daptomycin in 16 patients and linezolid in 1 patient based on physicians' judgement and clinical situations. MRSA was eventually eradicated in 12 of the 16 patients, and 7 of them had negative blood culture within 7 days of daptomycin treatment. Only two patients failed to achieve blood culture conversion under daptomycin, and 2 patients did not have subsequent blood culture collected after daptomycin use.

**Fig 1:**
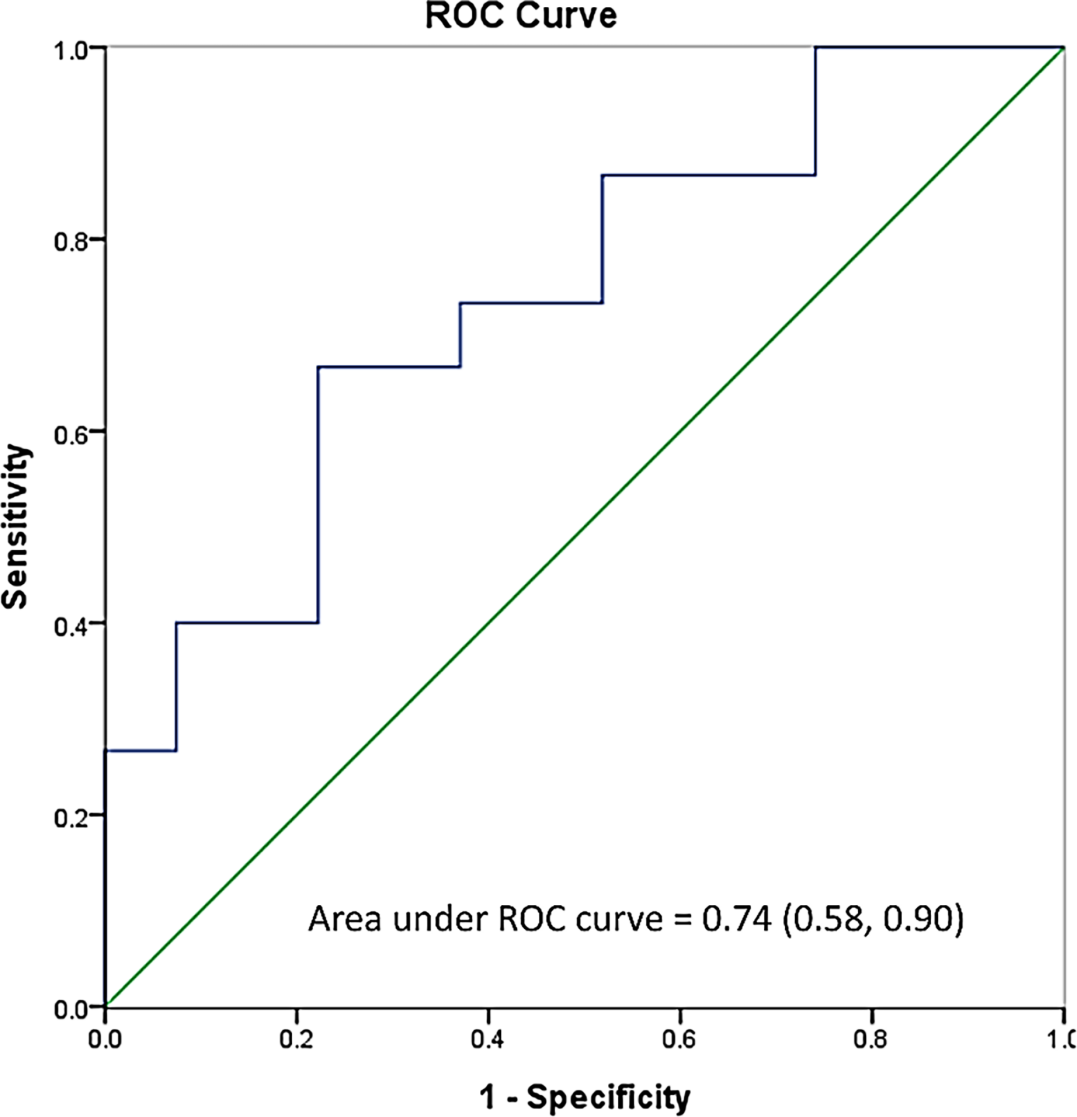
The receiver operating characteristic (ROC) curve of the C_pre-HD_/MIC at the cut-off value of 18.6 for the prediction of treatment outcomes. CI: confidence interval.

The prediction power of C_pre-HD_/MIC for treatment outcome was evaluated using the ROC curve analysis. The optimal cut-off value of C_pre-HD_/MIC was 18.6, with the sensitivity of 67% and the specificity of 78%. The area under the ROC curve was 0.74 (95% confidence interval: 0.58–0.90, *p* = 0.011) (Fig 1), and the positive predictive value and negative predictive value were 62.5% and 81%, respectively.

## Discussion

This is the first study known to the authors focusing on the correlation between the C_pre-HD_ of vancomycin and the treatment outcomes for MRSA bacteremia in dialysis patients. The relationship between vancomycin C_trough_, AUC_24_/MIC, and the clinical outcomes for MRSA infection has been well studied in the general population, and that C_trough_ of 15 mg/L or higher, corresponded to the AUC_24_/MIC > 400, was recommended by the IDSA guideline[2–7, 17, 18]. This standard was extrapolated for patients who received renal replacement therapy in clinical practice without adequate studies to explore the correlation between this target concentration and treatment outcome in this particular patient population. Until now, all relevant studies were designed to test if the dosing strategies would achieve the target C_pre-HD_ of 15–20 mg/L in patients who were on intermittent HD[12, 14]. However, it has not been demonstrated whether the specific target C_pre-HD_ can be translated into good clinical outcomes.

According to the results of our study, the success and failure groups did not have significant differences in the mean C_pre-HD_ as well as the proportion of patients with the C_pre-HD_ > 15 mg/L. However, after taking MIC into consideration, C_pre-HD_/MIC was significantly higher in the success group than that in failure group. Moreover, the ROC curve analysis suggested C_pre-HD_/MIC≧18.6 as an outcome predictor for treating MRSA bacteremia. Our data were also compatible with previous recommendations that the target level of vancomycin C_pre-HD_ should be 15–20 mg/L for a MIC of 1 mg/L[9]. As for the MIC distribution, around 70% of MRSA had vancomycin MIC value of 1 mg/L and pathogens with high MIC value (>1.5 mg/L) were almost distributed in treatment failure group, which was compatible with previous studies that high MIC level was a predict factor for poor treatment outcome.[19] However, it is difficult to distinguish to what degree is the failure caused by not attaining the PK/PD target and to what degree the higher MIC caused failure. Therefore, we excluded patients who suffered from pathogens with high MIC value (>1.5 mg/L), and the C_pre-HD_/MIC was still significantly higher in treatment success group than that in failure group (24.07 vs. 16.92, p = 0.04). This result demonstrated that attaining the optimal C_pre-HD_/MIC was important for treatment outcome of MRSA bacteremia in this population. Because vancomycin MIC crept in MRSA, which had high percentage of vancomycin MIC of 1 mg/L, and nephrotoxicity is generally not an issue in patients receiving HD, the aggressive vancomycin dosing strategy to achieve C_pre-HD_≧18.6 mg/L should be adopted before confirming MIC data[20].

Compared with a similarly designed study of vancomycin treatment in non-dialysis patients with MRSA bacteremia, the treatment success rate in this study was much lower (36% vs. 67.5%) despite of similar Charlson Comorbidity Index[4]. Two factors may explain the difference. First, the immune function is depressed in patients with renal failure. Functional abnormalities of monocytes, lymphocytes, neutrophils, and dendritic cells are directly linked with infection risk and treatment failure in this patient population.[8] Second, the formation of bacterial biofilm in dialysis patients may contribute to the high treatment failure rate. *Staphylococcus aureus* is the most common gram-positive organism involving in biofilm infection in dialysis patients [21, 22]. Some studies revealed that dialysis-related biofilm infections are difficult to eradicate as they developed antibiotic resistance[23–25]. Vancomycin has poor penetration into biofilm compared with daptomycin, and it is reasonable that the patients responded to daptomycin treatment in this study after vancomycin treatment failure.[26] Further studies are necessary to determine if daptomycin has better treatment effect than vancomycin in dialysis patients.

A multivariate analysis was performed by logistic regression. The covariates such as C_pre-HD_/MIC, Charlson Comorbidity Index, polymicrobial bacteremia, concurrent antibiotic use and septic shock were put into regression model, and found that C_pre-HD_/MIC was still the only significant variables to affect treatment outcome (OR:1.14, *p* = 0.013). Although more patients received immunosuppressants in treatment failure group than that in success group and this may confound our result, it did not put into regression model due to the extremely unbalanced distribution that patients received immunosuppressants all belonged to treatment failure group. Therefore, we tried to exclude these subjects and found that C_pre-HD_/MIC was still significantly different between the two groups (*p* = 0.035). This may further increase the strength of our conclusion that C_pre-HD_/MIC was a predictor for treatment outcome of MRSA bacteremia in renal failure patients receiving HD.

Based on our vancomycin dosing protocol for patients receiving HD three times per week, the proportion of C_pre-HD_ ≧ 18.6 mg/L was only 21%, which may have contributed to the relatively high rate of treatment failure. One possible cause for this low proportion of therapeutic C_pre-HD_ was that the loading dose used in the present study (<20 mg/kg) was too low. Vandecasteele et al. showed that a fixed loading dose of 20 mg/kg led to C_pre-HD_ ≥ 15 mg/L in only one-half of patients[9], supporting that a higher loading dose should be applied in order to achieve the optimal therapeutic C_pre-HD_.

There are several limitations of our study. First, the sample size was quite small in this study because of the low MRSA prevalence rate and MIC report has been available since 2013 in our institution. Further large prospective study is needed to confirm the treatment target provided by this study. Second, we only enrolled patients undergoing HD; this result may not apply to patients receiving other renal replacement therapy, such as peritoneal dialysis. Third, this was a retrospective study, and thus was not controlled for some confounding factors. Finally, overweight and obese patients were not represented in this study. Therefore, the result may not apply to this special population.

## Conclusions

We showed that C_pre-HD_/MIC was associated with the outcome of vancomycin treatment in dialysis patients with MRSA bacteremia. Targeting to achieve a C_pre-HD_/MIC of ≧ 18.6 may improve the treatment outcomes in renal failure patients who are on intermittent HD.

## Supporting information

S1 File Data set for analysis. (XLS)
